# MAITs and their mates: “Innate-like” behaviors in conventional and unconventional T cells

**DOI:** 10.1093/cei/uxad058

**Published:** 2023-05-31

**Authors:** Carl-Philipp Hackstein, Paul Klenerman

**Affiliations:** Peter Medawar Building for Pathogen Research, University of Oxford, Oxford, UK; Translational Gastroenterology Unit, Nuffield Department of Medicine, University of Oxford, Oxford, UK; Peter Medawar Building for Pathogen Research, University of Oxford, Oxford, UK; Translational Gastroenterology Unit, Nuffield Department of Medicine, University of Oxford, Oxford, UK

**Keywords:** MAIT cell, innate-like, unconventional, NKT, *T*_MIC_

## Abstract

Most CD4 and CD8 T cells are restricted by conventional major histocompatibility complex (MHC) molecules and mount TCR-dependent adaptive immune responses. In contrast, MAIT, iNKT, and certain γδ TCR bearing cells are characterized by their abilities to recognize antigens presented by unconventional antigen-presenting molecules and to mount cytokine-mediated TCR-independent responses in an “innate-like” manner. In addition, several more diverse T-cell subsets have been described that in a similar manner are restricted by unconventional antigen-presenting molecules but mainly depend on their TCRs for activation. Vice versa, innate-like behaviour was reported in defined subpopulations of conventional T cells, particularly in barrier sites, showing that these two features are not necessarily linked. The abilities to recognize antigens presented by unconventional antigen-presenting molecules or to mount TCR-independent responses creates unique niches for these T cells and is linked to wide range of functional capabilities. This is especially exemplified by unconventional and innate-like T cells present at barrier sites where they are involved in pathogen defense, tissue homeostasis as well as in pathologic processes.

## Teaser

T cells can recognize conventional (peptide-major histocompatibility complex) or unconventional ligands. In many cases, “unconventional” T cells such as MAIT cells also show innate-like features and can respond independently of their TCR. Recent data have indicated innate-like features in conventional T cells, such as the recently described *T*_MIC_population in gut.

## Graphical Abstract

**Graphical Abstract:**
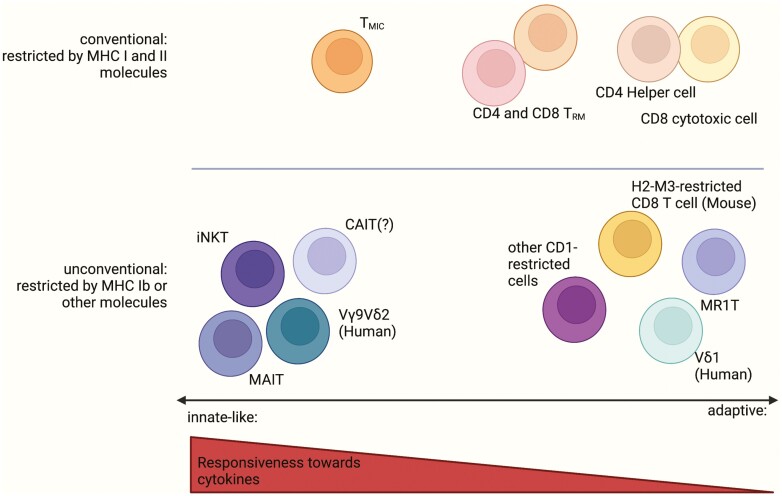

## Introduction

T-cell receptor (TCR)-bearing lymphocytes are one of the most prominent and versatile immune cell populations. Most T cells respond to peptide-antigens presented by conventional MHC I and MHC II molecules and display an adaptive immune behavior, allowing them to establish specific immune memory against their respective cognate antigens and the sources these are derived from. While these conventional, adaptive T cells play essential roles in a wide range of important immunological processes including cell-mediated cytotoxicity, immunological help and immune regulation, several T-cell populations that differ in the way they recognize antigen or function have also been discovered. These include cells interacting with unconventional antigen presenting molecules, expressing limited and semi-invariant TCRs and often displaying features of antigen-independent responses driven by cytokine sensing. It has been more recently observed that T cells with conventional restriction can also display innate-like features, as exemplified by the T_MIC_ (MHC II-restricted, innate-like and commensal specific T cell) population observed in human and mouse intestine. In other words, innate-like behaviors and unconventional restriction are not always directly linked. This review aims to summarize our current knowledge about unconventional and innate-like T cells, their functions, differences from and similarities to conventional T cells, focusing on the newest populations.

## Unconventional and innate-like T cells: two sides of the same coin?

**Figure 1.**
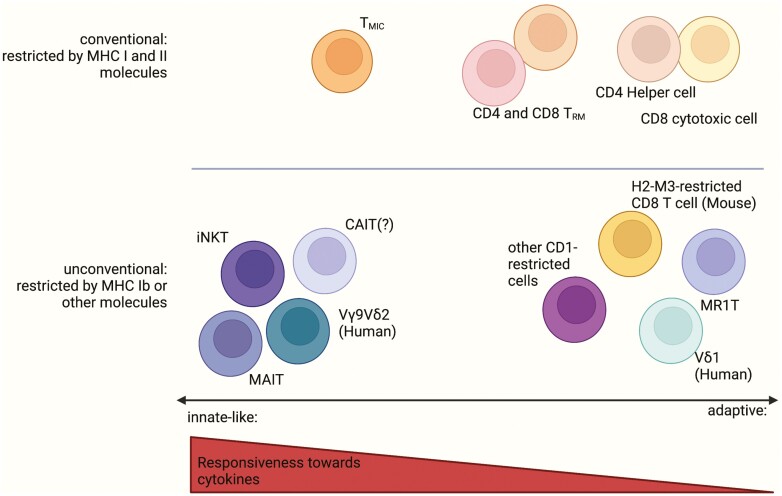
Unconventional and innate-like T cells: T cells can be grouped as conventional or unconventional based on their restriction molecules: conventional polymorphic MHC-molecules or unconventional non-polymorphic molecules. In contrast, the classification as adaptive or innate-like can be done along a gradient with conventional CD4 and CD8 T cells and well-established unconventional populations like MAIT, iNKT, and potentially CAIT cells on the respective ends of the spectrum. Some unconventional T cells including MR1T, certain γδ and CD1d-restricted T-cell populations display adaptive behaviour upon activation, while on the other hand conventional T cell can acquire innate-like features as illustrated by the T_MIC_ (MHC II-restricted, innate-like, and commensal-reactive) phenotype found in colonic microbe-reactive T cells (see below for description) or arguably by the bystander activation of tissue-resident (*T*_RM_) cells. This figure was created with Biorender.com.

There are two key features that distinguish some of the most well-known unconventional T-cell populations from conventional, adaptive T cells:

(A) Their TCRs recognize non-peptide antigens presented by unconventional antigen-presenting molecules and (B) they are able to mount immune responses in a cytokine-dependent fashion independently of their TCRs, in a manner reminiscent of innate immune cells and hence, in the scope of this review, termed innate like. In contrast to the strict dependency on unconventional antigen-presenting molecules, the degree to which cells display this innate-like capacities can vary between different cell subsets and can be more accurately described as a gradient (Fig. 1). This behaviour is typically associated with expression of the transcription factor PLZF (Promyeloic leukema zinc finger protein, *ZBTB16*).

### MAIT cells

In humans the most abundant cell type displaying these features are mucosal-associated invariant T (MAIT) cells. These cells are characterized by the expression of a semi-invariant TRAV1-2-TRAJ33/20/12 or TRAV1-TRAJ33 TCR in combination with a limited number of β-chains humans and mice, respectively, and recognize riboflavin-derived antigens presented by the MHC-Ib molecule, major histocompatibility complex, class I-related (MR1) [1, 2]. While the abundance of MAIT cells differs between different mammalian species [3–5], MR1 shows a remarkably high degree of conservation suggesting an important evolutionary function.

MAIT cells constitute a notable percentage of human T cells in blood, liver, and other barrier organs like the lung [6] and express a large number of different cytokine receptors enabling them to respond in an innate-like fashion to cytokines including IL-12, IL-18, IFN I, and TNF. This innate-like functionality allows MAIT cells to participate in immune responses even in the absence of their cognate antigen, including a range of viral infections and viral vector-based vaccinations [7, 8].

Transcriptionally, MAIT cells are regulated by a set of key transcription factors. These include T-bet and RoRγt, the archetypical transcription factors driving the production of TH1 and TH17 functions, respectively. Further, MAIT cells express high levels of PLZF, a transcription factor which is considered to be lineage-defining for unconventional, innate-like T cells [9].

MAIT effector functions span classic Th1 and Th17 cytokines [10], as well as cytotoxic effectors [11] and a follicular helper phenotype [12]. Consequently, roles for MAIT cells have been proposed in diverse contexts including anti-bacterial and anti-viral defence, tumor immunology, regulation of autoimmunity, and metabolic diseases [2, 13]. More recently, work from several groups also showed that MAIT cells are capable of promoting tissue repair *in vitro* and in the skin *in vivo*[14–17]. MAIT-mediated repair processes were induced by TCR-dependent triggering in most models analyzed up to date and were absent *in vitro* when MAIT cells were activated in a strictly TCR-independent fashion. This suggested the existence of separate adaptive and innate-like functional modules in MAIT cells which expression is regulated by the availability of the appropriate respective triggers and then would be integrated into cellular function. The study by du Halgoulet *et al.* [17] from 2023 through demonstrated the expression of a key repair factor, Amphiregulin (AREG), by MAIT cells in response to IL-18 without any apparent TCR signalling *in vivo*. Hence, while MAIT cells do express repair-associated factors in a TCR-dependent manner, different TCR-independent signals, or combinations of them can also possess this potential, at least in regarding individual effector molecules. This suggests that MAIT cells overall represent a key population in maintaining and repairing barrier tissues.

### iNKT cells

Natural killer T (NKT) cells were originally defined as T cells expressing molecules typically associated with NK cells, like NK1.1 in mice and CD161 or CD56 in humans. As these can be expressed by a large variety of both conventional and unconventional T cells, the modern definition of NKT cells was restricted to populations recognizing lipid antigens presented by the MHCIb molecule CD1d [18]. In mice, the majority of these cells belongs to an innate-like population termed type I or invariant NKT (iNKT) cells, defined by the expressing of a semi-invariant TRAV10-TRAJ18 TCR and are present in various tissues with a particular enrichment within the liver [18, 19]. In humans, iNKT cells are less frequent and express TRAV11-TRAJ18 [20, 21]. MAIT and iNKT cells show some striking similarities in terms of development, function, expression of PLZF and tissue distribution, but it is worth noting that iNKT cells seem to have a generally broader array effector functions spanning T_H_1, T_H_17, T_H_2, T_FH_ as well as regulatory phenotypes, while MAIT cells based on our current knowledge are restricted to T_H_1, T_H_17, and T_FH_ functions [10]. While human and murine iNKT cells recognize the murine-sponge-derived glycolipid antigen α-galactosyl-ceramide (α-GalCer) [22], as well as several bacterial derived lipid antigens via their TCR [23], they are also able to mount strong cytokine-mediated innate-like responses [24, 25]. Interestingly, while iNKT cells in general are well established and routinely described as innate-like population, there is evidence that they in fact require some degree of TCR engagement by low affinity ligands to get activated in a cytokine-dependent manner [26, 27]. Mouse models have established important roles for iNKT cells in cancer, infection, autoimmunity, and tissue homeostasis [28–30], while their roles in humans—due to their low abundancy—are less well defined.

### Innate-like γδ T cells

While all γδ T cells are unconventional in the sense that they recognize antigens independently of conventional MHC molecules [31], different subsets either with or without innate-like characteristics exist in both humans and mice.

A major set of human innate-like γδ T cells express a TRGV9-TRDV2 (Vγ9Vδ2) TCR [32, 33] reminiscent of the invariant TCRs expressed by MAIT and iNKT cells. The Vγ9Vδ2 TCR interacts with butyrophilin 3A1 (BTN3A1) [34–36] in the presence of host-derived prenyl pyrophosphate or microbial metabolites like 4-hydroxy-3-methyl-but-2-enyl pyrophosphate (HMB-PP) [37] constituting a unique form of indirect antigen recognition. Vγ9Vδ2 T cells are a relatively abundant cell population in the blood [38], can be found in tissues like the liver and duodenum [39], and are involved in anti-microbial responses [37, 40]. They parallel human MAIT cells transcriptionally [21, 41, 42] and phenotypically, expressing high levels of cytokine receptors, surface markers like CD161 and importantly transcription factors like PLZF [39]. As a result, they can mount strong cytokine-induced responses independently of their TCR [39].

In mice, PLZF-expression was reported in TRGV1-1-TRDV6-3 (Vγ1.1Vδ6.3) T-cells [43, 44] as well as in cells expressing the TRGV5 (Vγ5) and TRGV6 (Vγ6) chains [45]. Vγ1.1Vδ6.3 T-cells share striking phenotypically and transcriptionally similarities with murine iNKT and MAIT cells [46, 47] and localize preferentially to spleen and liver [48, 49]. Interestingly, the absence of PLZF-expression leads to a reduction of the functional capacities of Vγ1.1Vδ6.3 T cells but did not affect their total numbers [9, 43, 44]. In a similar manner, Vγ5 T cells, also known as dendritic epidermal T cells (DETC), which play important homeostatic roles in the skin [50, 51], do express PLZF, but do not depend on it for their development or ability to colonize the murine skin [45]. In contrast, PLZF is required for Vγ6 T-cell development and maturation [45]. This subset be found in the lung, the uterus, tongue, and liver [52] and are believed to be important contributors to IL-17-dependent immune responses.

### Other unconventional innate-like T cells

In addition to the major populations of MAIT, iNKT and Vδ2 T cells, several smaller T-cell subsets have been described that can be classified as both unconventional and innate-like. In 2011, Uldrich, Patel *et al.* identified a small subset of T cells expressing a public Vα10-Jα50 TCR in mice [53]. This TCR represents an alternative iNKT TCR, as, while allowing better responses to glucose-containing glycolipid ligands than the TRAV10 TCR, it recognizes α-GalCer presented by CD1d. Recently, a CD8-positive population of T cells expressing an invariant TRAV12-1*-*TRAJ6 TCR, termed Crohn’s-associated invariant T (CAIT) cells was found in the blood of Crohn’s disease patients [54]. Transcriptionally, CAIT cells clustered together with MAIT and iNKT cells and expressed high levels of IL18R, suggesting that they might also be able to mount TCR-independent innate-like responses. A follow-up study revealed that CAIT cells can recognize small sulfonate molecules presented by CD1d [55], firmly placing these cells inside the unconventional T-cell family.

## Unconventional cells lacking innate-like functionality

While innate-like behaviour is a key characteristic of MAIT and iNKT cells, it is not a feature shared with all T-cell subsets restricted by unconventional antigen-presenting molecules.

Several populations restricted by MR1, CD1 molecules, and others have been identified in humans and animals that can be distinguished from conventional, innate-like T cells by the lack of PLZF-expression and innate-like behavior, as well as the use of a diverse TCR-repertoire suggesting adaption to multiple different cognate antigens (Fig. 1).

### MR1T cells

Like MAIT cells, MR1T cells recognize antigens presented by MR1. However, in blood MR1T cells are considerably rarer than MAIT cells, lack PLZF-expression and are found in similar frequencies (<0.1%) as peptide-specific conventional T cells [56]. Further paralleling conventional T cells, MR1T cells were shown to express a broad range of different TCRs, and different clones were shown to differ substantially in their transcriptional phenotype [57] and functional profiles [56]. Some MR1T cells were found to respond to a large variety of tumor cells lines in a TCR- but not riboflavin-dependent manner and are currently being explored for their potential in cancer immunotherapy [56, 58, 59]. In addition, MR1-restricted T cells expressing different TCRs and responding to microbial antigens [60–62] have been described, pointing towards a very versatile role for MR1 as an immune sensor in different settings. While the targets of MR1T cells, at least for some of the known clones, are known, due to their overall low frequencies they are typically identified and studies by *in vitro* cloning approaches in humans. Thus, many aspects of their biology such their thymic development remain to be defined.

### Type II NKT cells

Type II NKT cells is an umbrella term summarizing all CD1d-resticted αβT cells not expressing the α-GalCer-reactive TRAV10-TRAJ18/TRAV11-TRAJ18 TCR. While some of these like CAIT cells are possibly innate like, many, also known as diverse NKT cells, are not. They are not as numerous in mice as iNKT cells but outnumber them in humans [63]. Several different subsets responding to different lipid antigens were described [64, 65] and accordingly type II NKTs express a larger range of different TCRs [64, 66]. Expression of PLZF is much lower in type II NKTs compared to iNKT cells [65] and different models have shown that their activation is mainly dependent on TCR-signals [25, 67, 68]. Type II NKTs were suggested to play regulatory roles in a range of different diseases including infections, cancer, and metabolic disorders [31, 65].

### H2-M3: restricted T cells

H2-M3 is an MHC Ib molecule present in mice but absent in humans that presents microbial and mitochondrial N-formulated peptide antigens [69]. H2-M3-resticted CD8 T cells participate in the immune response against *Listeria monocytogenes* [70–72], *Chlamydia pneumoniae* [73], *Mycobacterium tuberculosis* [74], and *Streptococcus epidermidis* [75]. In 2018, Linehan *et al.* provided a comprehensive characterization skin derived in H2–M3-restricted CD8 T cells, demonstrating the use of a diverse TCR repertoire and the lack of notable PLZF expression, distinguishing these cells from unconventional innate-like T cells like MAIT cells and iNKT cells. Functionally, H2-M3-restricted T cells can show diverse phenotypes [73, 75, 76] and *S. epidermidis*-specific H2–M3 CD8 T cells were shown to express tissue-repair-associated gene signatures, in line with the observation that healing of skin injuries is delayed in H2-M3^−/−^ mice.

### Adaptive γδ T cells

Comparisons between different γδ T-cell populations revealed that several of them show features also found in adaptive conventional T cells. In humans, blood-derived γδ T cells utilizing TRDV1 (Vδ1) and TRDV3 (Vδ3) chains cluster transcriptionally closer to conventional CD4 and CD8 T cells than to MAIT or iNKT cells [21], in line with the expression of molecules associated with naïve T cells [77]. Human Vδ1 and Vδ3 TCRs bind to a range of different antigens presented by CD1 molecules or directly interact with stress-associated MHC I-like molecules [31] and play roles in antiviral immunity [32] and tumor-surveillance [78]. An adaptive phenotype and behavior in response to HCMV infection was also reported for a subset of human Vδ2 T cells not expressing the Vγ9 chain associated with human innate-like Vδ2 T cells [33].

In mice, besides the PLZF-expressing TRGV1-1, TRGV6, and TRGV6 cells, a range of other subsets exists, often associated with particular tissues and specific effector profiles. These include intestinal TRGV7, dermal, lung, and hepatic TRGV4 T cells and other TRGV1 T-cell subsets [52, 79]. Not all ligands for the various murine γδ T-cell populations have been identified to date, but direct recognition of the murine stress-induced molecules and recognition of CD1d-presented antigens has been described [31].

### Group 1 CD1-restricted T cells

In addition to CD1d, humans express three more antigen-presenting members of the CD1-family: CD1a, b, and c [80]. CD1a-resticted T cells are relatively abundant in blood and skin and respond to a variety of antigens including squalene, wax esters, and skin oils [81–83], constituting an auto-reactive T-cell population. Functionally, these cells were shown to be producers of IL-22 [83] and are localized to the skin [31]. Auto reactivity was also observed in CD1c-restricted T cells and they have been specifically linked to tumour-derived lipid-antigens [81, 84]. CD1b-resticted T cells also recognize diverse lipid antigens and feature an overall diverse TCR repertoire, but subsets with a restricted or even-semi-invariant TCR usages have been described in LDN5 like [85] and germ-line encoded mycolyl-reactive (GEM) T cells [86] respectively. Interestingly, both subsets respond to mycobacterial lipid antigens and GEM T cells, despite the expression of a semi-variant TRAV1-2-TRAJ9 TCR, lack the expression of PLZF or NK-associated markers found in innate-like T cells [86].

## Innate-like T cells responding to antigen presented by conventional MHC molecules

Just as restriction to unconventional antigen-presenting molecules can be found in certain adaptive T-cell populations, certain T cells that would be considered conventional in terms of their antigen-restriction and TCR-usage turned out to display innate-like features in certain settings (Fig. 1).

### Tissue-resident memory T cells

Several lines of evidence show that murine memory T cells can mount antigen-independent responses under certain circumstances, although human memory T cells do this only to a very limited extent [87, 88]. This phenomenon, which is often referred to as bystander activation, is very prominent in CD8+ tissue-resident T cells (T_RM_), where it was shown in different mouse models comparing cells with defined antigen specificities [89–91] and different virus-specific memory populations in humans [91]. Bystander activation could be considered an innate-like activation mode as it does not require TCR signalling but instead is mediated via cytokines. In particular, IL-15 signaling was shown to play a crucial role mainly driving the acquisition of TCR-independent cytotoxic effector functions [91–93], while activation by IL-18, particular in synergy with IL-12 allows TCR-independent production of IFNg in *T*_RM_ cells to a much higher extent than in blood-derived memory cells [90, 91, 94]. A comparable innate-like behavior by CD4 *T*_RM_ is less well studied [91, 95], but nevertheless has been observed in mouse models [91] and recently in human Crohn’s disease patients [96]. It is noteworthy that particular CD4 T cells at barrier sites, e.g., intestine show high levels of key cytokine receptors like IL18R1 [97] supporting the idea that tissue-resident cells might be especially sensitive to cytokine signaling. Despite these characteristics, *T*_RM_ cells are usually not considered innate like as they lack several other features associated with established innate-like population, notably expression of PLZF.

### PLZF-Expressing MHC-Restricted T Cells

The innate-like phenotype of MAIT was shown to be linked to a specific transcriptional core program that is shared with related populations and cells displaying innate-like behaviour [21, 42]. Key features of this program aside from cytokine receptors (*IL18R1*, *IL12RB2*, and *IL23R*) include the expression of transcription factors like RoRγt and especially *ZBTB16*, encoding PLZF. It is noteworthy that PLZF expression has not been reported in the aforementioned memory cell subsets, separating them from MAIT cells, iNKT cells, and human Vδ2 cells. However, PLZF-expression in T cells not belonging to the established unconventional, innate-like T-cell populations were identified in the fetal human intestine [98], the murine thymus [99] and to a small degree within CD8 T cells expressing intermediate levels of CD161 and PLZF [87]. It is not well established what roles these subsets play in health and disease, but they all show increased capacities to produce effector molecules compared to other conventional T cells. Fetal human PLZF+ CD4 T cells produce large amounts of IFNγ and could be involved in perinatal inflammatory diseases and are enriched in the cord blood of newborns with gastroschisis. In contrast, conventional PLZF+ T cells from the murine thymus were described to express IL-4 and regulate the phenotype of CD8 T cells. Interestingly, both of these subsets seem to be linked to the presence of microbial antigens. Lastly, at least some human CD161^int^ CD8 T cells bind to MHC I-dextramers loaded with antigens derived from common viruses like CMV, EBV, or influenza and in general show higher expression of cytotoxic effector molecules like Granzyme B and Perforin compared to other CD8 T cells. Hence, CD161^int^ CD8 T cell might be particularly effective at dealing with viral infections.

#### T_MIC_ cells

Elevated PLZF expression was also recently shown in MHC II-restricted T cells in the murine and human colon [100]. There, it was specifically linked to T cells responding to antigen derived from commensals including *Escherichia coli* and *Candida albicans* in humans or bacterial flagellin in mice. Transcriptionally, these MHC II-restricted, innate-like, and commensal-reactive T cells (T_MIC_) cluster together with murine iNKT cells, share transcriptional signatures with human MAIT cells, and also display innate-like functionality in response to TCR-independent stimulation with cytokines. Interestingly, while human T_MIC_ cells express CD4 and, like other innate-like cells, comparatively high levels of CD161, the corresponding murine population does not express either the CD4 or CD8 co-receptor nor NK1.1. In addition to their MHC II-restriction, murine T_MIC_ cells express THPOK, the master regulator of CD4 lineage commitment, clearly showing that these cells are related to the CD4 lineage [100]. It is currently unclear which signals drive the development of T_MIC_ cells in either species and what is the reason behind the superficial differences, particular regarding CD4 expression, between humans and mice. However, over 80% of Cbir1 flagellin-specific T cells displayed a T_MIC_ phenotype in the colonic lamina propria, while in other tissues the majority of these cells had a conventional CD4+ phenotype. Similarly, human microbe-reactive CD4 T cells in the blood were shown to be relatively heterogenous in terms of CD161 and chemokine receptor expression [101] as well as a mixture of central and effector memory phenotypes, in contrast to the CD161^hi^ effector memory T_MIC_ cells in the human colonic lamina propria [100]. These data suggest that antigen-availability, potentially in combination with yet-to-be-identified local cues, plays a key role in bestowing innate-like properties onto these cells. Importantly, the human T_MIC_ gene signature showed only a weak correlation with several previously published gene signatures of T_RM_ cells [100] indicating that the T_MIC_ phenotype is not a necessary consequence the adaptation of memory T cells to peripheral tissues but rather represents a unique phenomenon restricted to certain subpopulations, possibly these responding to commensal microbes.

In line with the idea that T_MIC_ cells arise in response to various microbes, a diverse oligoclonal TCR repertoire can be found in both humans and mice.

Like MAIT cells [15–17], T_MIC_ cells can express tissue-repair associated factors like VEGF and Amphiregulin upon TCR-triggering *in vitro*, although they were also shown to possess strong pro-inflammatory capacities as illustrated by aggravated pathology in a murine DSS-colitis model and cells expressing key molecules of the T_MIC_ phenotype were present and showed signs of activation in human ulcerative colitis [100].

Given their large numbers, especially in the human colon (approx. 30% of all lamina propria CD4 T cells on average), further research is required to fully elucidate the developmental pathways leading to T_MIC_ formation, as well as their specific roles in both homeostasis as well as in infection and inflammation.

## Conclusion

While unconventional and innate-like properties go together in prominent populations like MAIT and iNKT cells, certain T cell subsets present as unconventional adaptive or conventional innate-like instead.

This on the one hand broadens the repertoire of antigens the immune system can screen for and allows for the formation of adaptive responses and immune memory based on lipids, metabolic components, and modified peptides in response to microbes and cancer. Innate-like features in conventional T cells on the other hand enable them to participate in a wider range of immune response even in the absence of their cognate antigen. Such non-specific activation could be helpful in containing pathogens by a larger initial innate response, but also has the potential to be harmful as illustrated by the detrimental effects of T_RM_ bystander activation in different models or the pro-colitogenic action of T_MIC_ cells in murine colitis. Future studies will be needed to elucidate the mechanisms driving these and to elucidate if they could be manipulated.

An interesting feature seen in several innate-like and unconventional T cells is the ability to contribute to tissue homeostasis and accelerate wound-repair. Several studies using MAIT cells have linked the expression of tissue repair-associated factors to TCR signaling [14–16], suggesting that promoting tissue repair results from an adaptive response. In line with this idea, wound healing by H2M3-restriced CD8 T cells in mice is accelerated after topic association with ligand-providing bacteria and reduced in H2M3^−/−^ mice [75] and repair-associated factors are expressed in T_MIC_ cells specifically upon TCR- but not cytokine triggering *in vitro* [100]. Additional work is needed to elucidate in detail how tissue repair by innate-like T cells is regulated however, as a more recent paper suggests that MAIT-dependent skin healing is independent of MR1 and does not require sustained TCR signaling [17]. Establishing which cytokines or combinations of cytokine can contribute to this important process and which factors specifically they can or cannot induce constitutes a key task for future studies in the field.

Another specific question important in this context and beyond it relates to the role of TCR signalling in innate-like T-cell activation. As mentioned above, in the context of certain infections iNKT cells require TCR signals from endogenous low affinity ligands to allow for their cytokine-dependent responses and in MAIT cells somewhat similar observations have been made in the context of SARS-CoV2 infection, where MAIT activation and responses are to some extent dependent on MR1 [102], even though the virus does provide any ligand. The surprisingly ubiquitous presence of riboflavin ligands [103] *in vivo* could provide an explanation for the latter finding. Further studies are required to establish whether what we consider “innate-like” behaviour is in fact to some degree enabled by low degree TCR-activation.

**Figure 2.**
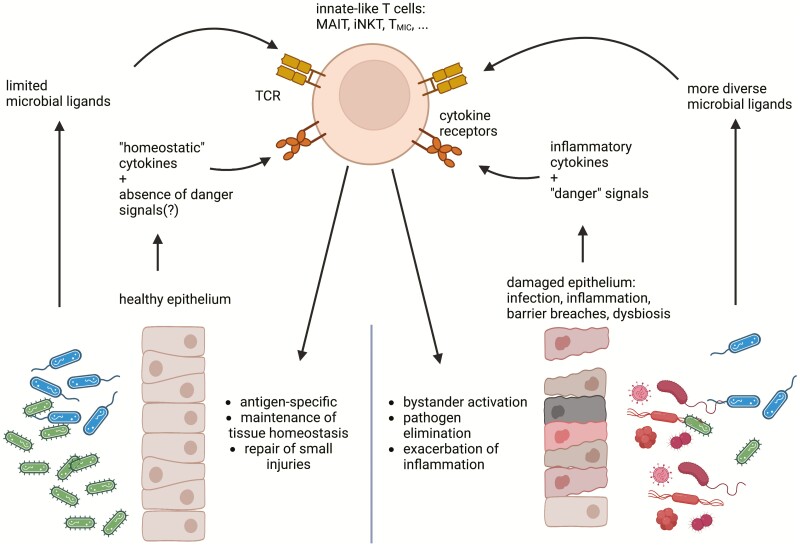
Innate-like T cells as guardians of barrier sites: The potential to express tissue repair-associated factors and to contribute to tissue regeneration has been described for several innate-like T cells populations including MAIT, γδ, iNKT and also in cells with the newly discovered *T*_MIC_ phenotype. This function seems to be particularly associated with the TCR-dependent activation pathway but can at least in part also triggered by individual cytokines like IL-18. The regulation of this function is not fully understood at the moment, but integration of these signals with additional input potentially provided by “danger”-signals like bacterial PAMPs or necrotic cell material could be crucial in determining whether a homeostatic or inflammatory response is initiated. While the former would contribute to maintenance and regeneration of barrier site, the later could play a key role in pathogen elimination and potentially exacerbating inflammatory conditions. This figure was created with Biorender.com.

Given the large numbers of MAIT or iNKT cells in the human or murine livers, γδ T cells in the skin and T_MIC_ cells in the colonic lamina propria, innate-like T cells have a large potential to play an important role in guarding barrier and tissue integrity (fig. 2) and hence, understanding the mechanisms regulating their different functions could open-up new avenues for therapeutic interventions in the future.

## Data Availability

No new data were generated for this manuscript.

## Abbreviations

CAIT cell: Crohn’s-associated invariant T cell
GEM cell: germ-line encoded mycolyl-reactive T cell
MAIT cell: mucosal-associated invariant T cell
MHC: major histocompatibility complex
MR1: major histocompatibility complex, class I-related
iNKT cell: (invariant) natural killer T cell
PLZF: promyeloic leukema zinc finger protein
*T*_MIC_: MHC II-restricted, innate-like and commensal-specific T cell
*T*_RM_: tissue-resident memory cell
TCR: T-cell receptor
ZBTB16: zinc finger and BTB domain containing 16.
